# An Investigation of Mental Health, Social Isolation and Loneliness Among Older African Migrants in Australia During COVID-19

**DOI:** 10.3390/healthcare14142185

**Published:** 2026-07-20

**Authors:** Daniel Doh, Mahesh Chougule, Teddy Nagaddya, Samuel Kwabena Dakey, Sanjeev Dahal, Sipho Sibanda

**Affiliations:** 1 Department of Social Work and Social Policy, The University of Western Australia, Perth 6009, Australia; daniel.doh@uwa.edu.au; 2 Research Unit on Contemporary Challenges: Social Policy Impact on Social Mobility and Development, Faculty of Social Administration, Thammasat University, Bangkok 10200, Thailand; 3 School of Social Sciences, Western Sydney University, Sydney 2747, Australia; t.nagaddya@westernsydney.edu.au; 4 The Marcs Institute, Western Sydney University, Sydney 2747, Australia; 19970077@student.westernsydney.edu.au; 5 Discipline of Social Work, The University of Notre Dame Australia, Sydney 2008, Australia; sanjeev.dahal1@nd.edu.au; 6 Department of Sociology, University of Pretoria, Pretoria 0028, South Africa

**Keywords:** African migrants, Australia, connectivity, coping, loneliness, mental health, social isolation, strategy, older people

## Abstract

Background/Objectives: Social isolation, loneliness, and mental health challenges among older people have been a global problem long before the advent of COVID-19. In Australia, there are many reports of how COVID-19 has worsened older people’s mental health, social isolation and loneliness experiences. However, the narratives have focused on the general population with little attention being paid to migrants from minority groups who have traditionally relied on informal social networks. Methods: In this study, we employed community-based participatory research, supported by thematic analysis, to investigate how 21 older West African migrants in Greater Sydney experienced social isolation and loneliness during the COVID-19 pandemic and the coping strategies they adopted. Results: The findings show a growing trend in weakening social connectivity, exacerbated by COVID-19, resulting in significant social impacts, mental health concerns, and daily lifestyle changes. There is a greater concern about mental health experiences, including fear, anxiety, confusion, and depression. Participants noted forming new habits, such as watching movies and learning new digital technological skills, as the main coping strategies, which enabled them to connect with churches and other organisations. However, their coping strategies adopted were provisional, unsustainable, and uncoordinated. Conclusions: There is a need for locally government-supported activities that foster and sustain social interaction, as well as more community-led and locally supported initiatives that can alleviate social isolation and loneliness during global pandemics.

## 1. Introduction

Long before COVID-19, social isolation and loneliness were major concerns for older people globally due to their detrimental effect on mental health and well-being [1]. Several studies have documented the problem’s extensive repercussions in middle-income countries and advanced economies [2,3,4]. The challenges of social isolation and loneliness are worse for older migrants, especially those from minority groups, due to reasons such as shrinking social networks [5], language and integration difficulties [6], and overall fragilities associated with ageing [7], which minimise the ability to make social connections. Coincidentally, the advent of the COVID-19 global pandemic has offered another unprecedented challenge to social isolation and loneliness among older people [4], including migrants who are believed to bear the greater brunt [8,9]. In Australia, there are many reports of the ways the COVID-19 pandemic has worsened the social isolation and loneliness experiences of older people [10,11,12]. However, little is known about how older migrants from culturally and linguistically diverse (CALD) backgrounds, such as those from African backgrounds, have experienced social isolation and loneliness during COVID-19.

Conceptually, social isolation and loneliness are twin terms often used together, but they mean different things. Wu explains social isolation as a person’s withdrawal from others and having few interactions and social contacts within a community [13]. It is a state of feeling rejected by one’s community or close relations [14,15]. Other studies explained social isolation as an objective state of having few social relationships or infrequent social contact with others [16,17,18]. Loneliness, on the other hand, concerns the subjective feeling associated with being socially isolated. Olofsson, Rämgård recounted that loneliness among older people is best understood as the state of awareness of being completely separated from society and close relations [19]. Luo and Waite explained loneliness as a distressing feeling that occurs when the social relations of the individual are inadequate in the older generation [20].

The triple jeopardy of being a migrant, an older person, and belonging to a minority cultural group presents a higher risk of social isolation. In a Swiss study, Ehsan, Bolano [21] observed the struggles of older migrants in creating a sense of belonging, leading to higher expressions of social isolation compared to mainstream older people. Pike and Crocker recount the relationship between social isolation, migration, and ageing, noting that migrants suffer from the risk of weaker family bonds in their new location, which also leads to social isolation among the individual [15]. Similar findings were made by Murenje and Sibanda on Zimbabwean migrants living in Johannesburg, South Africa [22]. In a Canadian study, Gierveld, Van der Pas noted that immigrants have been reported to be lonelier than natives who speak their original language [23]. Sibanda et al. argued that COVID-19 influenced individuals’ social connectedness with family, friends, and communities, impairing the relationships’ continuity [24]. Given COVID-19’s global impact, it has become more critical to explore older migrants’ social isolation and loneliness more generally, while also attempting to understand how being a migrant minority interacts with social isolation and loneliness during the pandemic.

There are three main reasons why it is important to pay attention to discussions of social isolation and loneliness among older migrants globally, especially Africans in Australia, during the COVID-19 pandemic. Firstly, social isolation and loneliness present tangible physical and mental health challenges to older people [25,26,27], such as the risk of chronic stress, cardiovascular diseases [28], and dementia [29], which, if not attended to, undermine Goal 3 of the Sustainable Development Goals that focus on promoting the health and well-being of all people. Second, migrants from CALD backgrounds are more likely to be at risk of social isolation and loneliness [30,31] and need greater attention; third, there is growing evidence that the strong traditional collectivist culture and intergenerational solidarity of Africans are dissipating within the Australian diaspora. Therefore, to reduce inequality and promote their well-being, it is imperative to explore the lived experiences of older African migrants during the COVID-19 compulsory lockdowns and social distancing rules and examine ways to support and strengthen their social interaction regardless of any future pandemic. This paper aims to understand isolation and loneliness through the lived experience of older African migrants and examine the coping strategies they adopted to address them during COVID-19 pandemic in Sydney, Australia. The main research questions that guided the study were the following: What are the lived experiences of social isolation and loneliness among older African migrants in Australia during the COVID-19 pandemic? What coping strategies do they adopt?

## 2. Materials and Methods

### 2.1. Setting and Study Design

In this study, we used a community-based participatory research (CBPR) approach [32,33,34] supported by thematic analysis to investigate social isolation and loneliness experiences of a cross-section of older West African migrants (65 years and above) living in Greater Sydney, Australia. The combination of these two methods provided an opportunity for an in-depth view of the data [34] and strengthened the analysis of social isolation and loneliness, and how these are interpreted within broader social and community structures [35]. The study was conducted among Ghanaian, Sierra Leonean, and Nigerian communities in Sydney, Australia, with support from an African not-for-profit organisation. The CBPR component of the design involved engagement and collaboration between the researchers and the community-based organisations to promote collaborative community knowledge production and capacity building [36]. In line with the CBPR model, we established an inter-community project advisory committee (IPC) comprising eight-member community leaders to provide an oversight role and enhance engagement, participation, and collaboration in the co-production of knowledge of social isolation and loneliness. The IPC was specifically responsible for community entry, identification of community members at risk of isolation, attending committee meetings to discuss research questions, field progress discussions, and validation of preliminary findings.

### 2.2. Recruitment

Through collaboration with the IPC, we purposively sampled 21 older African migrants (seven from each of the three participating communities) who were at risk of social isolation and loneliness as determined by the committee. The older African migrants were recruited from three African communities in Sydney, namely, Ghanaians, Sierra Leoneans, and Nigerians. To be eligible for the study, participants had to meet the following inclusion criteria: be at least 65 years old at the time of the interview, be of African descent, be a resident in NSW during the COVID-19 lockdown and be identified by the community as at risk of social isolation and loneliness. In exceptional cases, community members were also allowed to include a community member who was more than 60 years old and less than 65 years old at an extreme risk of isolation and loneliness. Community leaders first identified and contacted potential respondents and solicited contact information, which was subsequently passed on to the Chief Investigator (CI). The CI then invited participants by telephone to participate in the study. Once contacted, participants confirmed their availability and willingness to participate in the study; they were asked to provide a preferred date and time and were later visited by a community-based research assistant for the interview. The in-depth interviews were face-to-face and were conducted either in the local language of the older person or in English, based on the respondent’s preference.

### 2.3. Data Collection

We used in-depth interviews with each identified participant as a conversational approach to facilitate the sharing and analysis of lived experiences using an interview guide collaboratively designed with the IPC through an interactive manner predicated on community knowledge and the extant literature on the COVID-19 lockdown and older people. The interview guide captured two key areas: (1) participants’ experiences of social isolation and (2) coping strategies during the COVID-19 lockdowns. The interview guide also contained questions to explore participants’ opinions on the public health measures implemented locally, including those impacting the older African migrant community. All interviews were conducted by trained community research assistants identified by the IPC between May and September 2021.

**Figure 1:**
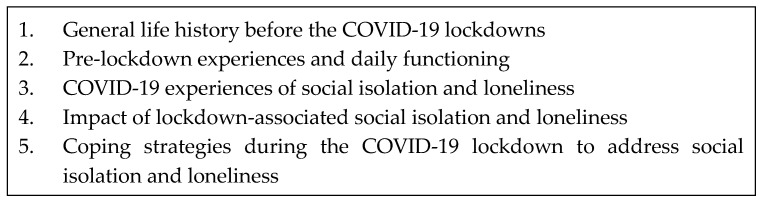
Interview guide.

We obtained informed consent from all participants before the interviews. The informed consent process involved reading a printed summary of the study’s aims and potential learning opportunities of the study in the research community and outlining the rights of participants, including confidentiality and withdrawal from the study. After explaining our data handling and confidentiality protocols, we obtained permission from participants to audio-record their interviews. To maintain confidentiality, we replaced the names of participants with the first letter of their country of origin and a number indicating when the interview was completed. For example, G3 indicates the third participant interviewed from Ghana, one of the three African countries from which participants originated. We removed other potentially identifying information of participants from the transcripts. We encrypted and saved all audio recordings, transcripts, and participants’ personal information on a password-protected computer and followed other Standards for Reporting Qualitative Research guidelines [37]. The interview guide is depicted in Figure 1.

### 2.4. Data Analysis

We transcribed the audio-recorded interviews verbatim, and two members of the research team, including the CI, analysed them using NVivo 13 software. Two research team members used NVivo to initially code and analyse the data following a thematic analysis approach [38]. We developed an analytical framework with two sections: (1) a section of pre-existing deductive codes derived from previous studies that explored the COVID-19-related experiences of older adults [39] and (2) an open-ended section of inductive codes emerging from each participant’s experience. We created cases for each study participant in NVivo from the data and used the cases to capture socio-demographic characteristics of participants referenced as attributes by NVivo 13. We coded the transcripts guided by the deductive nodes listed in the analytical framework and coded according to inductive nodes emerging from the transcripts. The analysis team was primarily interested in understanding shared experiences based on the stories of each participant. The team later identified and coded meaningful quotes from the transcripts into nodes that captured quotes on lived experiences of social isolation and loneliness as distinct parent nodes. We labelled quotes under broader nodes after reviewing each quote and its description. Based on the participant’s story, we organised the generated nodes into the two reportable themes [40], namely, (i) lived experiences of social isolation and loneliness and (ii) coping strategies for social isolation and loneliness. After organising the nodes and sub-nodes, we explored patterns between attributes of participants and their responses coded under experiences and coping strategies as the final stage of the analysis. The researchers implemented multiple strategies to strengthen the quality, trustworthiness, authenticity, and credibility of the qualitative data. These strategies encompassed ongoing reflexivity, the maintenance of a detailed audit trail, member checking to verify interpretations, peer debriefing to enhance analytical rigour, and the provision of rich, thick descriptions to support contextual depth.

For example, as a predominantly African-led research team, with several members originally migrating from Ghana, we were conscious of the potential for our Africanness to shape how we understood and interpreted the phenomenon. To mitigate possible biases arising from our positionality as Africans living in Sydney and experiencing the COVID-19 lockdown, we engaged non-African colleagues on the team and employed CBPR processes. This reflexive approach helped ensure that our interpretations were balanced and reflective of diverse perspectives.

Ethical clearance for the study was obtained from the institutional review board of Western Sydney University (H14219). Throughout the research process, the researchers remained attentive to the ethical implications of methodological and procedural decisions. In alignment with the core ethical principles outlined by Strydom, several measures were implemented to safeguard participants’ well-being [41]. Participants were informed of their right to withdraw from the study at any stage without a penalty or negative consequences. All participants received a clear explanation of the study’s purpose and voluntarily agreed to take part. No deception was employed, and no incentives or promises of payment were offered. The participants provided written informed consent. The letter of informed consent detailed the aims of the study and the nature of participation. The researchers ensured that no coercion, pressure, or misleading information was used during the consent process. Confidentiality and anonymity were upheld using codes in place of participant names. All research materials were securely stored in a password-protected folder that only the research team had access to.

## 3. Results

### 3.1. Demographic Profile of Participants

The demographic profile of participants was constructed from the analysis of information that pertained to their age, gender, average stay in Australia, employment, religion, and marital status. The demographic profile is presented in Table 1.

### 3.2. Experiences of Participants During the COVID-19 Lockdown

Participants reported that COVID-19 mitigation measures, such as the city lockdown, significantly affected their social networks, daily routines, physical and mental health, and finances. Analysis of data shows that many experienced heightened social isolation and loneliness during the period. The findings show that 11 of 21 participants described experiencing social isolation only, mostly characterised by not being part of things, not meeting people, fewer social interactions, and feeling withdrawn from community and family. Only two participants reported feelings of loneliness, which they described as a sense of separation even when others were present, limited or absent opportunities for meaningful conversation, and the experience of living entirely alone. Notably, five participants experienced both social isolation and loneliness. Only three participants did not report either condition. From the data, only three participants did not report either social isolation or loneliness. Although some participants experienced social isolation before COVID-19, the situation worsened during COVID-19-related lockdowns, leading to strong feelings of loneliness. Other emerging themes include reported changes to activities of daily life, mental health experiences, financial experience, and general physical health. Table 2 describes the emerging themes and their associated descriptions.

#### 3.2.1. Experiences of Social Isolation and Loneliness During the COVID-19 Lockdown

Of the 21 participants, 11 reported social isolation only, 2 reported loneliness only, 5 experienced both, and 3 experienced neither. According to most participants, the COVID-19 lockdown, particularly the restrictions to movement, visitors and physical distancing, led to feelings of social exclusion, isolation, and loneliness among themselves as older African migrants. They were also isolated from their families and support systems. G6 (female) shared, “*Socially, I was isolated from many people, including my own children, grandkids and my friends. For instance, one of my children and her husband used to visit us during the lockdown, but I did not allow them to enter my house. They waved to greet me from their car parked in front of my house. I talked to them through my window*”.

In some instances, participants living with their family members in the same house stopped making some forms of intimate contacts such as hugging and close contact. N2 (male) shared, “*Oh yes, great change, a great difference. During the lockdown, we were not allowed to go out, we were isolated, serious isolation, and for me, as an elderly person, I felt as if I was segregated from others, I was not free to move around like before and was categorised as one of the vulnerable ones. The fear of COVID was very real to me. Even in the house, I stopped hugging my family members, for me, I was so afraid to go outside for exercise*”. Some participants reported that the feeling of loneliness was popular among older African migrants, particularly those who are divorced, or single and those living alone, as expressed by G1 (67 years, female), “*My friends (age mates) have expressed the same feelings of loneliness and social isolation during the lockdown. Those who were heavily affected were the single or divorced. It is worse when nobody, like kids and family members, is around you to converse with you*”. Consistent with the notion that marital status and family connections modulate loneliness among older African migrants during the lockdown, most of the participants who admitted feeling lonely were either single or divorced and living alone. The lonely feeling left some participants wishing that they were back home in Africa, where, according to them, they would be less lonely compared with their lockdown experience in Australia: “I wish I were back in my native country because here nobody converses with you due to the nature of this society…and probably my colour (race).” “*I feel so lonely and isolated due to the lockdowns, but it wasn’t the same before the pandemic*”, G4 (66 years, female). For other participants, having other family members around did not mitigate the loneliness. A participant recalled being unintentionally aggressive towards his daughter because of the frustrations of the lockdown “*Yes, my family was with me, but it wasn’t really helping the feeling. You know, I was not myself. At a point, my daughter complained I was becoming aggressive; it was just the nature of the lockdown that was getting to me.*” G4 (66 years, female).

#### 3.2.2. Daily Life Impacts of the COVID-19 Lockdown

Participants stated that the COVID-19 lockdown changed their daily routines. N6 (female) lamented, “*Before the lockdown, I could visit the hairdresser to get my hair done, but during the lockdown, I could not do that for a while, and as a woman, that did not feel good*”. Participants described many adjustments to lifestyle, school, work, training, exercise, hobbies and other routines during the COVID-19 lockdown. For some participants, the lockdown presented challenges to their mode of schooling and working: “*I am currently studying, and we moved online, which was not very good because a teacher is supposed to see the state of a student and vice versa. All the things you learn and enjoy so much were all gone in a twinkle of a moment*”. Other participants, such as S4 (female), worked from home during the lockdown: “*I am part of the workers at logistics who take care of the documentation. During the lockdown, my work changed, so I worked from home*”. Other participants gave up shopping and other lifestyle activities: “*I felt restricted from getting to do what I love, shopping. I was always in the house and went out only to attend to my clients as a disability care professional*”, shared G5 (female). Most participants commenced walking and other routine exercises within permitted distances during the lockdown. For some participants, such as N4 (female), these routine walks constituted significant changes during the lockdown: “*I also go for walks between 40 min to 1 h almost every day, and it helps. During the lockdown, I made some of these changes because it was beneficial to me*”.

#### 3.2.3. Mental Health Experiences of the COVID-19 Lockdown

Participants shared several mental health experiences brought by the COVID-19 lockdown. For example, S6 (female) stated, “*I spoke to my friends, and they all feel lonely and socially isolated. Some are going through depression and mental health-related problems*”. Participants reported experiencing anxiety, depression, confusion, being fearful and aggressive because of the COVID-19 lockdowns. For participants such as G4 (66 years, female), the inability to physically communicate with family members was depressing: “*I slept a lot, and I felt bored and depressed because there was no one around me to talk to me. I spoke to family members and my children on the phone, but as you are aware, you cannot substitute physical interactions with online conversations*”, while G5 (female) recalled, “*I felt lonely and fearful of the possibility of dying or alienation from reality. I always thought and dreamt of the afterlife when I die more than any other thing during the compulsory lockdown*”. The lockdown-related restrictions resulted in a combination of unhealthy feelings of sadness, loneliness, and confusion. For example, N7 (female) recounted, “*I could not see my other children and grandchildren. This really made me sad and lonely. It was not easy for me to understand why family members could not see each other. At some time, I started wondering if my children were deliberately avoiding me. It took a lot of explaining by my daughter and son-in-law to fully understand what was happening*”. For some participants, the lockdown coincided with other challenging life events like sickness, death or hospitalisation of family members. For instance, G4 (66 years, female): “*I was experiencing hernia, stomach ulcer and depressive disorders before COVID-19 started. The pandemic has worsened these conditions. I don’t go out, and I am always in my room. I am not able to go for a walk because I am afraid that I may contract the virus. I sleep a lot, and I feel bored and depressed because there is no one around me to talk to. I speak to family members and my children on the phone, but as you are aware, you cannot substitute physical interactions with online conversations*”.

#### 3.2.4. Financial Experiences of the COVID-19 Lockdown

The COVID-19 lockdown had positive and negative financial impacts on participants through increasing or decreasing the amount of money they earn and spend. Participants such as N1 (male) lost their jobs and faced financial difficulties because of the COVID-19 lockdown: “*I got another job with a Private Hospital in Ashfield afterwards. I had been working for 3 months before COVID-19 broke out, and the department was closed down, leaving me without a job. I had to revert to Centrelink* (the leading Australian social insurance agency for supporting vulnerable community members) *to start getting a senior’s pension because of my age*”. Similarly, G4 (66 years, female) explained, “*The pandemic has derailed my abilities to go to work and negatively affected my finances. I was on government support for years, but it has ceased, and I need to work but I can’t due to the lockdown*”. For other participants, the lockdown did not affect their earning, it increased their spending: “*Although, the COVID-19 lockdown did not affect my job, it unleashed economic hardships on me, and I spent more than how I used to spend before the lockdown*” S4 (female). Participants experienced the impact of COVID-19 on finances differently, other participants such as G5 (female) admitted “*although, the overall impact of the pandemic has been negative on me, it came with some positive effects. During the COVID-19 lockdown, I was able to save a lot of money because I was spending less on food and other expenses on social events”.* Similarly, S3 (male) indicated that “*The COVID-19 lockdown affected my work by reducing the number of hours I worked, but at the same time, I was able to save money*”.

#### 3.2.5. Physical Health Experiences of the COVID-19 Lockdown

The physical health of some participants deteriorated because of the lockdown. For some participants, such as G6 (female), the lockdown constraints particularly restricted movements, affecting their health: “*Also, it affected my movement with compulsory restrictions and wearing of face masks, which limited my breathing capacity and health*”. For most participants, the lockdown’s impact on their physical health coincided with deterioration in their mental health resulting from the lockdown: “*As I said, I was not connecting well with my family, and then gradually I started feeling fatigued, and because of the lockdown, I could not see my GP. He later directed me to the hospital. While I cannot categorically say I was depressed, my inability to go out and meet people the way I used to put considerable stress on my mind and body, which resulted in my health deteriorating. Eventually, I was admitted to the hospital for 4 weeks and by the time I was discharged, the restrictions had started easing.*” N1 (male). Other participants reported gaining weight and feeling unwell because of limited movements during the lockdown: “*Oh yeah, too lonely, the loneliness was too much. You know it is not good when one just eats, feels lonely and just sleeps. Wake up again, have another meal and sleep. I became very fat, which was not good for my health. We are supposed to be moving about, but we did not have the right environment for that.*” N2 (male).

### 3.3. Coping Strategies Adopted by Older African Migrants in Australia

As mentioned earlier, participants reported that the COVID-19 lockdown affected their social networks, daily lifestyle, physical and mental health, and their finances. To mitigate these challenges, participants highlighted a number of coping strategies that they adopted. The reported coping strategies included daily life adjustments, including routine exercises (indoors and outdoors) and working from home, staying socially connected, online and media engagements and faith-based practices. A combination of some coping strategies was health-limiting (e.g., eating and sleeping), while most strategies encouraged positive adjustment to exercise, and a sense of spirituality, physical wellness and social connectedness. The coping strategies highlighted by participants are presented in Table 3.

#### 3.3.1. Social Connections as Coping Strategies During the COVID-19 Lockdown

Participants indicated that initiating social connections is one of the strategies they used to cope with the COVID-19 lockdown. G5 (female), recounted, “*I was isolated from my friends physically, but I made contact with people via the phone to reach out when I felt lonely*”. Participants connected physically and remotely to friends, families, and other acquaintances during the lockdown. For physical in-person connections, one of the coping strategies by participants, such as S3 (male), was interacting with family members: “*I was not lonely and socially isolated. I had my wife and children around me, so we chatted and watched movies together as a way of coping*”. Most participants connected with people remotely through varied forms of communication, including phone calls, text messages, video calls, and emails. “*I have also been calling people back home (Nigeria) just to chat and share. Lack of social interaction makes me feel lonely anyway because normally it was not like that before.*” N4 (female). Few participants interacted with people while keeping a safe physical distance during the lockdowns. For example, G1 (age 67, female) shared her experience, “*I couldn’t come into physical contact with my friends and church members. My case was worse because I was also battling an injury. Friends who wanted to come to my house for a visit and chat with me were restricted. Those who came close to my house had to stand far away from my window and send greetings as well as food and cash. They were prevented from entering the house*”.

#### 3.3.2. Online and Media Engagements as Coping Strategies During the COVID-19 Lockdown

Some participants stated that, as a way of coping with the COVID-19 lockdown, they spent some time online and on social media platforms. G1 (age 67, female) stated, “*I became addicted to my TV, mobile phone and social media. The TV gave me information about the COVID-19 cases around the world. I was constantly in touch with people on the phone, including my family members within and outside Australia. In addition, I was involved in online prayer meetings with fellow Christians. YouTube and WhatsApp gave me up-to-date news and funny videos about what is going on in the world, especially my home country, Ghana*”. Most participants regularly accessed media content like videos and audios on social media platforms during the lockdown as a way of coping with the restrictions of the lockdown. Particularly, funny videos were popular among participants who accessed social media platforms during the lockdown: “*I watched African movies. The dramatic and documentary movies. In addition, I was sleeping and relaxing with social media funny videos.*” G7 (female). Most participants attended Church services through the internet: “*I was not lonely and socially isolated. I have my wife and children around me, so we chatted and watched movies together as well as attended church online.*” S3 (male).

A closer examination of how older men and women coped with social isolation and loneliness during COVID-19 indicates notable gendered patterns in preferred strategies. As shown in Table 4 below, women were substantially more likely than men to rely on watching television or movies as their primary coping mechanism (15 women compared with 4 men). In addition, social media engagement emerged as the second most common strategy used more frequently by women (10 participants) than men (2 participants). Telephone conversations were the least utilised strategy for both groups. While these findings are meaningful, they are not unexpected given the considerable difference in sample sizes between male and female participants in the study.

#### 3.3.3. Adapting and Adjusting Daily Life Changes as Coping Strategies During the COVID-19 Lockdown

Participants reported adjusting and adapting their daily lives to cope with the lockdown. Daily adjustments were made by participants strategically to feel more normal, purposeful or restful in their daily life. Daily routines included regular walking, hobbies, working from home and online schooling. For some participants, walking was a way of keeping well during the lockdown: “*I walk around my house twice every day. After my morning Bible devotional readings, I step out of my house and walk from one end of my street to the end. I do the same in the evening.*” G1 (67 years, female). Other participants picked up new habits: “*During the lockdown, I started reading a lot of books and reading articles online to keep abreast with what is going on.*” G1 (67 years, female). Some participants attributed their ability to deal with the lockdown to working during the lockdown: “*I felt there’s always something to do, even if I didn’t have anything to do besides work, there is something to do to keep me busy, as there’s always something for me to do to keep me busy. So, throughout the isolation from the lockdown, I kept myself very busy with work, and that kind of helped me through that phase until we were able to gradually start meeting again.*” N3 (male).

#### 3.3.4. Faith-Based Coping Strategies During the COVID-19 Lockdown

Most participants were inclined towards spirituality during the lockdown; some participants attributed their ability to deal with the lockdown to God. Most participants, such as N4 (female), had inspired spiritual notions of life during the lockdown from the Bible: “*I was scared and slowly withdrawing from everything. Eventually, I drew courage from my faith as a Christian, particularly resting on the part of the Bible where it says we have not been given a Spirit of fear, so I urged my wife, and we started going back to church after the restriction was eased. She was really concerned, even after the restrictions were eased for my health, but I assured her we were not going to contract the disease. Since then, we have been going to church on Sundays and Tuesdays, and we have participated in different church activities since then*”. Most participants remotely participated in church services and worship sections. For some participants, church activities were a way of dealing with the boredom associated with the lockdown: “*I attended church programs online to take away my boredom and disconnection from the world.”* S4 (female). Other participants watched Christian programming and listened to worship music with scripture. N4 (female) recounted, “*Sometimes I would pick up my phone to watch sermons from pastors, sometimes I listen to songs on my phone, or listen to news from Nigeria, and so on to keep me busy. The children also keep me busy, too, anyway, so I thank God*”. Some participants “*used the lockdown to draw closer to God by reading the Bible extensively.*” G6 (female).

## 4. Discussion

The findings of this study demonstrate that COVID-19 mitigation measures, particularly prolonged lockdowns, had far-reaching consequences for older African migrants, affecting their social networks, daily routines, physical and mental health, and financial stability. Although some degree of social isolation existed before the pandemic, the restrictions introduced during COVID-19 significantly intensified these experiences. The high proportion of participants reporting social isolation and those experiencing loneliness or both simultaneously underscores the compounded vulnerability of this group.

The emotional language used by participants, such as “*depressed*”, “*anxious*”, “*fearful*”, “*excluded*”, “*worried*”, “*segregated*”, and “*isolated*”, illustrates the depth of psychological distress associated with the disruption of their social worlds. These expressions align with the broader literature documenting the mental health impacts of pandemic-related social isolation on older adults [26,42,43], but they also reveal the unique pressures faced by minority migrant groups who rely heavily on informal, culturally grounded support networks [44].

The intensification of social isolation during the lockdown suggests that public health measures, while necessary for infection control, inadvertently exacerbated pre-existing social vulnerabilities. For older West African migrants, whose social connections often extend beyond the household and are embedded in community, religious, and cultural practices, the sudden loss of face-to-face interaction had profound consequences. The findings, therefore, highlight the need to consider cultural and structural factors when assessing the social impact of pandemic responses.

There have been several studies documenting social isolation and loneliness in the general population of older people during COVID-19, with some reporting worsening quality of social relationships [44,45,46]. However, only a few studies have explored social isolation and loneliness among migrants during the pandemic. In a study on Chinese migrants in Canada, the authors found social isolation and loneliness to be a serious concern, given that these older migrants were completely alienated from mainstream social interaction, fuelled by racism and ageism during the pandemic [47].

Before the onset of COVID-19, there had been reported cases of social isolation and loneliness among migrants from different contexts. Chen and Schulz [48] and Khosravi and Rezvani [49] stated that a large number of older people whose children are domiciled in other countries join them, and the available evidence from the United Nations and other reputable international organisations maintains that this number has increased recently. The children of such older immigrants spend less time on their social needs, and this leads to depression and social isolation. The phenomenon is common among the parents of the first generation of immigrants who bring their parents to the new destination. Another study reported that due to ageing, living in foreign lands like New Zealand can be challenging with little or no opportunities because of resettlement and ill health. In Australia, Ip and Lui discovered that elderly Chinese migrants are less involved in social networks and social activities due to their age and decrease in family size compared to the large family size and connections in their native country, China [50]. Older people among the Chinese immigrants experience a greater sense of neglect, isolation and rejection, particularly beyond the age of 70 years. Similar findings were made by Doh et al. regarding the experiences of older Ghanaians living in the cities of Australia and Ghana [51]. Also, language barriers pose a great challenge as older Chinese migrants find it difficult to abandon their native language and learn new ones. This phenomenon worsens the state of isolation of older people because it is hard to communicate with people beyond their families. All these studies are consistent with the current findings from this study.

One of the key findings of this study is the relationship between faith-based institutions and practices and the mitigation of social isolation and loneliness among older migrants. As the data show, many participants relied on faith-based practices, mainly through Christian churches, as coping mechanisms to address these challenges. Practices such as prayer, meditation, engagement with scripture, and participation in online church services provided meaningful support. Previous scholarship has similarly demonstrated that faith-based practices can positively influence experiences of social isolation and loneliness [52]. Other studies further report that religious faith and belief in God are associated with lower levels of loneliness and social isolation, reinforcing the significance of spiritual engagement as a protective factor [53].

Furthermore, recent studies have revealed that older migrants in other developed economies are susceptible to vulnerable realities, social embeddedness, and little care. Older migrants are also likely to experience loneliness and social isolation compared to non-migrants. Research concentrating on loneliness among older people is also likely to establish the diminishing familial bonds between the old and the young, as well as the families that the older migrants have moved to stay with. In the end, loneliness and social isolation lead to an increased rate of depression, mortality, coronary heart disease, and stroke.

Lastly, the study identified a clear gender-related trend in the ways older adults managed social isolation and loneliness during the COVID-19 pandemic. Compared with men, women were significantly more likely to use watching television or movies as their main coping strategy. Furthermore, engagement with social media was the second most frequently reported coping mechanism and was also more common among women than men. Nevertheless, because the study included a substantially larger number of female participants than male participants, these observed gender differences should be interpreted with caution and warrant further investigation. Supporting the need for additional research, Di Donna et al. reported that both social and scientific attention to gender-based violence during COVID-19 was notably limited, particularly in relation to the abuse of older adults [54].

Overall, the study contributes to a growing body of evidence showing that COVID-19 disproportionately affected socially marginalised groups. It also raises important questions about how future public health strategies can better account for the social and emotional needs of older migrants, whose experiences of isolation and loneliness may be shaped by intersecting factors such as migration history, cultural expectations, and limited access to formal support systems.

This study is subject to several limitations that should be considered when interpreting the findings. First, the relatively small sample size constitutes an important constraint. Although qualitative research does not seek statistical generalisation, a larger and more heterogeneous sample could have captured a wider spectrum of experiences, service contexts, and regional differences among older African migrants in Australia. The limited number of participants restricts the diversity of perspectives represented and may therefore reduce the extent to which the findings can be transferred to the broader population. Future research would benefit from replicating and extending this work using larger, more diverse samples that include participants from varied geographic locations and sociocultural backgrounds across Australia. Such efforts would strengthen the robustness, transferability, and policy relevance of the findings.

The recruitment process also presents a potential source of bias. Participants were accessed through community leaders. This relationship may have influenced participants’ willingness to openly discuss negative experiences. As such, the data may underrepresent critical perspectives on experiences during the COVID-19 period. Despite these limitations, the study offers important insights into the mental health, social isolation, and loneliness experienced by older African migrants during COVID-19. It highlights key structural and social challenges, as well as opportunities for improving culturally responsive support services. The findings, therefore, provide a valuable foundation for future research and policy development aimed at enhancing the well-being of this population.

## 5. Conclusions

The findings of this study reaffirm that social isolation and loneliness among older people are longstanding global concerns, but the COVID-19 pandemic intensified these challenges and brought renewed attention to their impact, particularly for older migrants. For older African migrants, the pandemic not only weakened existing social connections but also introduced significant emotional, psychological, and lifestyle disruptions. Participants described experiences of fear, anxiety, confusion, aggression, and depression, underscoring the depth of the mental health burden during this period.

Although participants adopted various coping strategies, such as developing new routines, engaging in solitary leisure activities, and acquiring digital skills to maintain contact with faith communities, these strategies were largely temporary, fragmented, and insufficient to address the broader structural issues underpinning their isolation. The study highlights the ongoing vulnerability of minority migrant groups, whose reliance on informal social networks made them particularly susceptible to the social consequences of the pandemic.

In moving beyond COVID-19, there is a clear need for more sustainable, coordinated, and culturally responsive interventions. Community-led initiatives, supported by local government, are essential for fostering meaningful and lasting social engagement. Strengthening digital literacy among older migrants also holds promise for enhancing social connectedness and reducing isolation. Ultimately, addressing social isolation and loneliness among older African migrants requires long-term, collaborative efforts that prioritise inclusion, accessibility, and community empowerment.

## Figures and Tables

**Table 1:** Demographic profile of participants (*n =* 21).

*Age*	
Average	67 years
Oldest	78 years
Youngest	62 years *
*Gender*	
Female	15
Male	6
*Average length of stay in Australia*	
Ghana	31.2 years
Nigeria	20 years
Sierra Leone	15.1 years
*Employment*	
In some form of employment	7
Not in any employment	14
*Disability*	
Has a disability	2
Has no known disability	19
*Religion*	
Christianity	21
*Marital status*	
Married	11
Widowed	5
Divorced	5

* This participant did not meet the selection criteria on age (65 years), but the IPC considered the individual to be at an extreme risk of social isolation and loneliness because of a disability.

**Table 2:** Summary of experiences of older adult migrants during the COVID-19 lockdown (*n =* 21).

Categories	Description	References from Nvivo Coding
*Experiences of lockdown*		
Social isolation	Feeling of exclusion (not being part of things), not meeting people, experiencing fewer social interactions, feeling withdrawn from community and family.	43
Loneliness	Feeling separated even with people around, limited opportunities for human conversation or connection, living alone.	18
Daily life changes	Changed daily lifestyle, self-care, sleep pattern, hobbies, work, exercise and school routines.	25
Mental health experiences	Frequently felt depressed, anxious, fearful, worried.	21
Financial experiences	Changed regular spending and saving patterns and financial behaviour, including reducing spending or creating financial hardships.	17
Physical health experiences	Lockdown-induced illnesses, including hospitalisation, and weight gain.	5

**Table 3:** Coping strategies of older African adults in the sample, COVID-19 Lockdown Study (*n =* 21).

Categories	Description	References from Nvivo Coding
Social connections	Contacting/reaching out to and communicating with family, friends, partners, and others through in-person interactions, or by phone, text message, or video call.	24
Online and media engagements	Watching movies and TV shows (not the news), using social media platforms (e.g., WhatsApp, Facebook, Twitter, YouTube).	19
Daily life changes	Engaging in hobbies (e.g., reading, writing, board games, arts and crafts, puzzles, crochet and knitting, games), working.	11
Faith-based practices	Taking part in religious activities (e.g., prayer, reading scripture, watching, or participating in faith services).	9

**Table 4:** Gender and coping strategies of older African adults in the sample, COVID-19 Lockdown Study.

Theme	Male	Female
Social media	2	10
Telephone conversation	2	6
TV and movies	4	15

## Data Availability

The datasets presented in this article are not readily available because of ethical restrictions.
